# Supplementary material for: Targeted next-generation sequencing of candidate regions identified by GWAS revealed SNPs associated with IBD in GSDs
Source: Front Vet Sci. 2025 Aug 6;12:1648911. doi: 10.3389/fvets.2025.1648911 (PMC12366469; doi:10.3389/fvets.2025.1648911)
Supplement: Supplementary file 4 [file Table_4.docx]

## Supplementary Table 4: BAMStat reports for the target region on Chr 9 and 11

Mapped-reads coverage report for target region on Chr 9 (Cases).

Mapped-reads coverage report for target region on Chr 9 (Controls).

Mapping report for target region on Chr 9 (Cases).

Mapping report for target region on Chr 9 (Controls).

Mapped-reads coverage report for target region on Chr 11 (Cases).

Mapped-reads coverage report for target region on Chr 11 (Controls).

Mapping report for target region on Chr 11 (Cases).

Mapping report for target region on Chr 11 (Controls).
