# Supplementary material for: Targeted next-generation sequencing of candidate regions identified by GWAS revealed SNPs associated with IBD in GSDs
Source: Front Vet Sci. 2025 Aug 6;12:1648911. doi: 10.3389/fvets.2025.1648911 (PMC12366469; doi:10.3389/fvets.2025.1648911)
Supplement: Supplementary file 5 [file Table_5.docx]

## Supplementary Table 5: Variants on chromosome 9

Variants within genes (high and moderated impact) and within 1 Kb up- and downstream gene (modifier impact) in the case population.

Hom Ref: sites with reference allele (AA), Het: Heterozygous (AB), Homo: Homozygous (BB), P value: Fisher’s exact probability test two tailed p value, Hardy-Weinberg equilibrium: HWE, HWE P: chi-square probability test p value used to test for HWE.

Variants within genes (high and moderated impact) and within 1 Kb up- and downstream gene (modifier impact) in the control population.

Hom Ref: sites with reference allele (AA), Het: Heterozygous (AB), Homo: Homozygous (BB), P value: Fisher’s exact probability test two tailed p value, Hardy-Weinberg equilibrium: HWE, HWE P: chi-square probability test p value used to test for HWE.

Variants overlapped between the case and the control populations with the same alternate in the both populations.

Hom Ref: sites with reference allele (AA), Het: Heterozygous (AB), Homo: Homozygous (BB), P value: Fisher’s exact probability test two tailed p value, Hardy-Weinberg equilibrium: HWE, HWE P: chi-square probability test p value used to test for HWE.
